# Outcome of MRI-guided vacuum-assisted breast biopsy – initial experience at Institute of Oncology Ljubljana, Slovenia

**DOI:** 10.2478/v10019-012-0016-0

**Published:** 2012-05-11

**Authors:** Marta Zebic-Sinkovec, Kristijana Hertl, Maksimiljan Kadivec, Mihael Cavlek, Gasper Podobnik, Marko Snoj

**Affiliations:** 1 Department of Radiology, Institute of Oncology Ljubljana, Ljubljana, Slovenia; 2 Department of Surgery, Institute of Oncology Ljubljana, Ljubljana, Slovenia

**Keywords:** breast cancer, MRI, MRI guided vacuum assisted biopsy

## Abstract

**Background:**

Like all breast imaging modalities MRI has limited specificity and the positive predictive value for lesions detected by MRI alone ranges between 15 and 50%. MRI guided procedures (needle biopsy, presurgical localisation) are mandatory for suspicious findings visible only at MRI, with potential influence on therapeutic decision. The aim of this retrospective study was to evaluate our initial clinical experience with MRI-guided vacuum-assisted breast biopsy as an alternative to surgical excision and to investigate the outcome of MRI-guided breast biopsy as a function of the MRI features of the lesions.

**Patients and methods.:**

In 14 women (median age 51 years) with 14 MRI-detected lesions, MRI-guided vacuum-assisted breast biopsy was performed. We evaluated the MRI findings that led to biopsy and we investigated the core and postoperative histology results and follow-up data.

**Results:**

The biopsy was technically successful in 14 (93%) of 15 women. Of 14 biopsies in 14 women, core histology revealed 6 malignant (6/14, 43%), 6 benign (6/14, 43%) and 2 high-risk (2/14, 14%) lesions. Among the 6 cancer 3 were invasive and 3 were ductal carcinoma in situ (DCIS). The probability of malignancy in our experience was higher for non-mass lesion type and for washout and plateau kinetics.

**Conclusions:**

Our initial experience confirms that MRI-guided vacuum-assisted biopsy is fast, safe and accurate alternative to surgical biopsy for breast lesions detected at MRI only.

## Introduction

Magnetic resonance imaging (MRI) is a method for the detection of many cancers.^1^,^2^ Contrast-enhanced magnetic resonance imaging (CE-MRI) is currently the most sensitive additional imaging method for the detection of invasive breast carcinoma and seems to be able to detect ductal carcinoma in situ (DCIS), especially high grade DCIS without necrosis.^3^ Compared with studies of MRI performance published in the 1990s, the specificity of breast MRI has gradually improved, mostly because of improved technology and increased reader experience.^4^

Suspicious MRI detected lesions are not always detectable by mammography and ultrasound. Positive predictive value for lesions detected by MRI alone ranges between 15% and 50% and it depends upon patient selection and MR interpretation algorythm.^5^–^8^ MRI guided procedures (needle biopsy, presurgical localisation) are mandatory for lesions visible at MRI only, when they look suspicious and have potential influence on therapeutic decision.^9^ MRI-guided vacuum assisted biopsy (MR-VAB) was pioneered by Sylvia H. Heywang-Köbrunner and first described in 1999.^10^

Since then, the technique has been optimized and its reproducibility proven in an European multicenter study.^11^ Some other authors have published their experience with this method and also with other vacuum devices.^12^,^13^ In 2006 there was an interdisciplinary consensus conference in Nordestedt, Germany.^14^ The purpose of this meeting was to determine technique and optimize quality assurance protocols. MR-VAB was first time performed at our institution in 2008 and since then it has become routinely used in our practice.

The aim of the present retrospective study was to evaluate the initial clinical experience with MRI-guided vacuum-assisted breast biopsy as an alternative to surgical excision and to investigate the outcome of MRI-guided breast biopsy as a function of the MRI features of the lesions.

## Patients and methods

### Patients and lesions

The selection criteria for the MR-guided biopsy were the presence of Breast Imaging Reporting and Data System (BI-RADS) 5, BI-RADS 4 and BIRADS 3 lesions that were visible by CE-MRI only. Initially, fifteen patients with 15 lesions had been referred for MR-VAB. In 1 patient the lesion could not be reproduced on the preinterventional planning MRI because of the technical problem. The median age of patients was 51 years (range, 35–71 years). In 6 patients mammography performed before biopsy showed dense breast or benign changes, in 2 patients asymmetry was described, in three cases there was architectural distortion only in one projection, in two cases mammographic and MRI findings were discordant and one patient was too young to perform mammography. In 7 patients sonography performed before biopsy failed to reveal a sonographic correlate and in 4 others sonography and MRI findings were discordant. In 3 patients sonography was not performed at the discretion of the radiologist because the possibility of identifying a sonographic lesion was presumed to be very low.

### MRI findings before biopsy

Except the MRI examinations of 2 women who underwent MRI at another institution, breast MRI was performed using a 1.5-T magnet (Magnetom Avanto, Siemens Medical Solution, Erlangen, Germany) with a dedicated bilateral breast surface coil with a prone position. The imaging protocol and parameters were as follows: axial T1-weighted image (TR/TE, 593/13) and short time inversion recovery (STIR) (TR/TE/TI, 12390/76/130) of both breasts were obtained with 3 mm slice thickness. Next, T1-weighted images were acquired using a 3D fast low-angle shot pulse sequence (FLASH) through both breasts (TR/TE 7, 8/4, 72, flip angle 25°). Precontrast images were obtained over a 313 × 448 matrix in the axial plane with a slice thickness of 1.0 mm with distance factor 20% before administration of the contrast agent. Then, contrast-enhanced dynamic imaging was performed with an injection of 0.1 mmol per kilogram of body weight of gadopentetate dimeglumine (Magnevist, Schering, Berlin, Germany); five sequential contrast-enhanced images were acquired at every 1 min 23 s. The precontrast images were then subtracted from the corresponding postcontrast images on a pixel-by-pixel bias with the use of the standard software subtraction function available on our console. Two radiologists reviewed all imaging studies in consensus. The BI-RADS MRI lexicon was used.

### MRI-guided biopsy

MRI-guided vacuum-assisted breast biopsy was performed using a 1.5-T magnet (Magnetom Avanto, Siemens, Erlangen, Germany) with MRI-supported Breast Immobilization and Biopsy System with the 4-channel breast coil (Noras MRI products GmbH, Höchberg, Germany) in prone position. Marker Block filled with diluted gadolinium contrast agent was used for reference point. The positioning device has medial and lateral compression plates for moderate compression.

An axial T1-weighted images were acquired using a 3D FLASH through both breasts (TR/TE 7, 6/4, 72, flip angle 25°). Precontrast images were obtained over a 256 × 320 matrix in the axial plane with a slice thickness of 1.0 mm with distance factor 20% before administration of the contrast agent. Twenty seconds after contrast agent had been injected; another axial T1-3D FLASH sequence was performed. 0.1 mmol per kilogram of body weight of gadopentetate dimeglumine (Magnevist, Schering, Berlin, Germany) was injected with a rate of 2 ml/s using an automated injector (CT/MRI injector Mississippi, Ulrich medical, Ulm, Germany). The volume of interest was selected to include the compression device and a Marker Block.

Biopsies were performed with a 9-gauge MRI compatible vacuum-assisted biopsy device (ATEC, Suros Surgical Systems, Indianapolis, USA). CAD stream diagnostic and interventional guidance tool, Version 4.1 (Confirma, Washington, USA) was used to target coordinates for biopsy. An axial T1-3D FLASH sequence was performed to control the needle placement.

The biopsy device was then placed into the breast through the coaxial needle. Multiple samples were obtained by turning the needle clockwise and around its axis. The biopsy site was marked with a titanium clip (ATEC TRI mark TD 13 – MR Biopsy Site Marker). “Postclip” axial 3D FLASH was performed to assess clip deployment. After biopsy, the breast is compressed with ice, sterile strips are applied, and a sterile gauze bandage is applied.

### MRI–guided wire localization

The procedure for wire placement was the same as that for breast biopsy. After localizing the lesion, a cannula with a wire loaded into it was inserted to the required depth. After confirming the position on axial images, the wire was placed into the lesion and the position was verified with a repeat axial scan. The wire gives a thin linear magnetic susceptibility artefact, and the lesion can be seen near the tip of the wire. It was then fixed with a lock at the skin surface to prevent dislodgement. The same positioning device for lesion localization with an MRI-compatible needle with a hooked wire, made of a special alloy for easy and safe penetration of the solid tumour tissue (TULOC, Somatex, Germany) were used. It has a diameter of 0.95 mm and it is 90 mm long. The wire was fixed into the lesion with the hooks at the end of the wire and onto the skin with a lock which prevents any dislodgement of the wire.

### Indication for MRI and MRI-VAB

The indications for MRI were classified into a screening setting and a diagnostic setting.

The indications for (MR-VAB) were:

MRI only seen lesions - targeted » second look« ultrasound and/or mammography showed normal or benign findings or findings that were not concordant with MRI findings.

Ultrasound was not performed at the discretion of radiologist, because the probability that lesion would be seen by ultrasound was very low.

### Management, follow-up, data collection, analysis

The MR-VAB was presumed to be adequate if imaging performed immediately after biopsy showed a cavity that unequivocally included the area of highest suspicion. Two view mammograms were also performed after biopsy to confirm clip localization. Surgical follow–up was recommended for all malignant lesions, for one high-risk lesion, and in one case, when the benign lesion was judged to have pathology result that was discordant with imaging findings. 6 of the 6 malignant lesions and 1 of the 2 high-risk lesions were operated upon at our institution and confirmed by operative histology. In 4 cases breast ablation and in 3 cases sentinel node and occult lesion localization (SNOLL) under X-ray guidance and surgical excision was performed. The reference standards were core biopsy results and postoperative histology. Medical records were reviewed for patient age, personal history of breast cancer, indication for MR mammography, radiologic results, histopathologic results and follow-up results. Histopathologic results were examined on the basis of pathologic report of MR-VAB and postoperative histology. The probability of malignancy for an MRI abnormality was calculated as the ratio between the number of lesions with pathologically proven malignancy and the number of biopsied lesions. We investigated the probability of malignancy for MRI abnormalities according to the MRI features of the lesions.

The retrospective study was carried out according to the Helsinki Declaration.

## Results

The biopsy was technically successful in 14 (93%) of 15 women. Median age was 51 years (range, 35–71 years). In 1 woman the biopsy was deferred because of a technical problem.

Fourteen lesions underwent biopsy, among them 4 lesions were categorized as BI-RADS 5, 8 lesions as BI-RADS 4 and 2 as BI-RADS 3. The median size of these 14 lesions was 2 cm (range, 0.8–6 cm). Among those 14 lesions, 4 were located between lower quadrants, 2 in lower outer quadrant, one between outer quadrants, 3 in upper outer quadrant, 3 between upper quadrants and one in upper inner quadrant. For 13 of the 14 lesions a single skin incision was made; for one lesion, a second incision was required for repositioning the stylet before biopsy. The median number of specimens obtained per lesion was 8 (range, 4–17). In 11 lesions, only a single round of tissue acquisition was necessary, in 3 lesions the MRI after the first round of tissue acquisition did not ensure lesion sampling, and a second round of tissue acquisition was performed. Clip placement was attempted in 14 lesions and was successful in 13 (93%). The median time to perform MRI-guided vacuum-assisted biopsy, from the original axial localizing images to the final images obtained after clip deployment was 39 min (range, 28–60 min). A complication was encountered in 1 of 14 patients (7%). The complication was a clinical haematoma.

Cancer was found in 6 of 14 lesions and in 6 of 14 women, based on the review of vacuum-assisted biopsy and surgical histology. Six were benign lesions and 2 were high-risk lesions. In two cases MR-VAB was considered uncertain according to the correlation of imaging and histopathology, therefore surgical biopsy and re-biopsy were performed.

**FIGURE 1:**
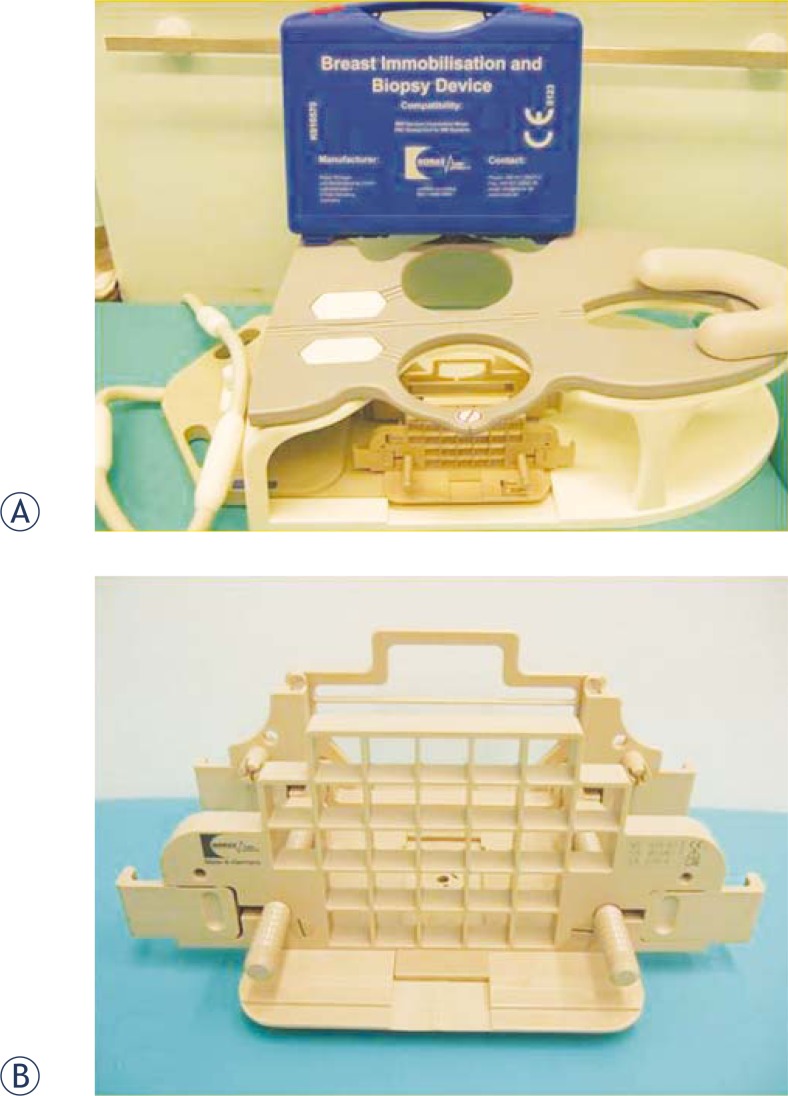
**A,B.** Biopsy coil device. Photographs show a four-channel breast biopsy coil with positioning device (A) and a grid-positioning device (B).

**FIGURE 2:**
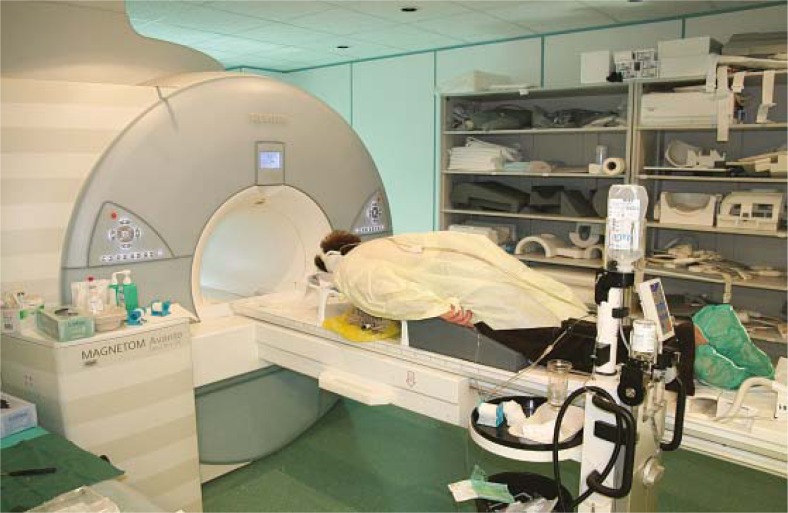
1.5 T Magnetom Avanto (Siemens, Erlangen, Germany). Patient lie face down on the exam table. Breast is moderately compressed in the mediolateral direction.

**FIGURE 3A–D:**
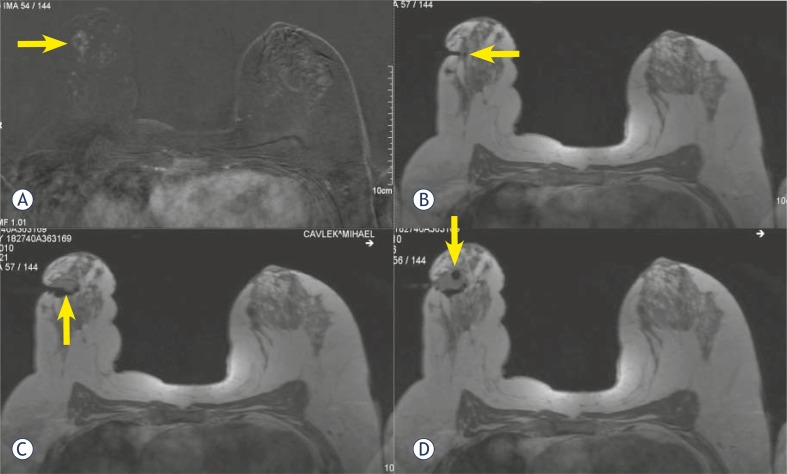
MRI-guided biopsy using axial, contrast-enhanced FLASH 3D T1W images. The pre-biopsy fat-suppressed image (A) shows an enhancing lesion in the right breast. The biopsy needle (B) is seen after localization, along with the lesion. Following the biopsy (D) the small magnetic susceptibility artefact due to the clip in situ is seen.

**FIGURE 4:**
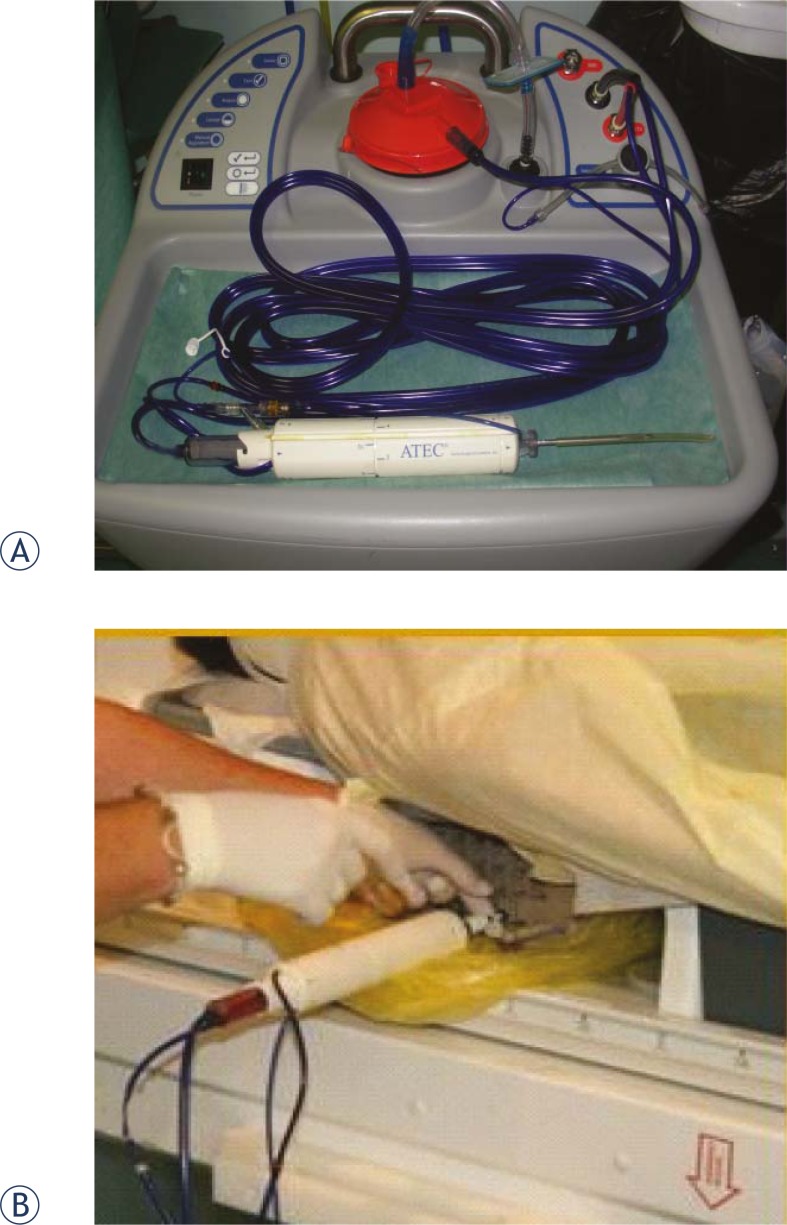
MRI-guided biopsy device (a) is inserted into breast to acquire tissue specimens (b)

**FIGURE 5A–C:**
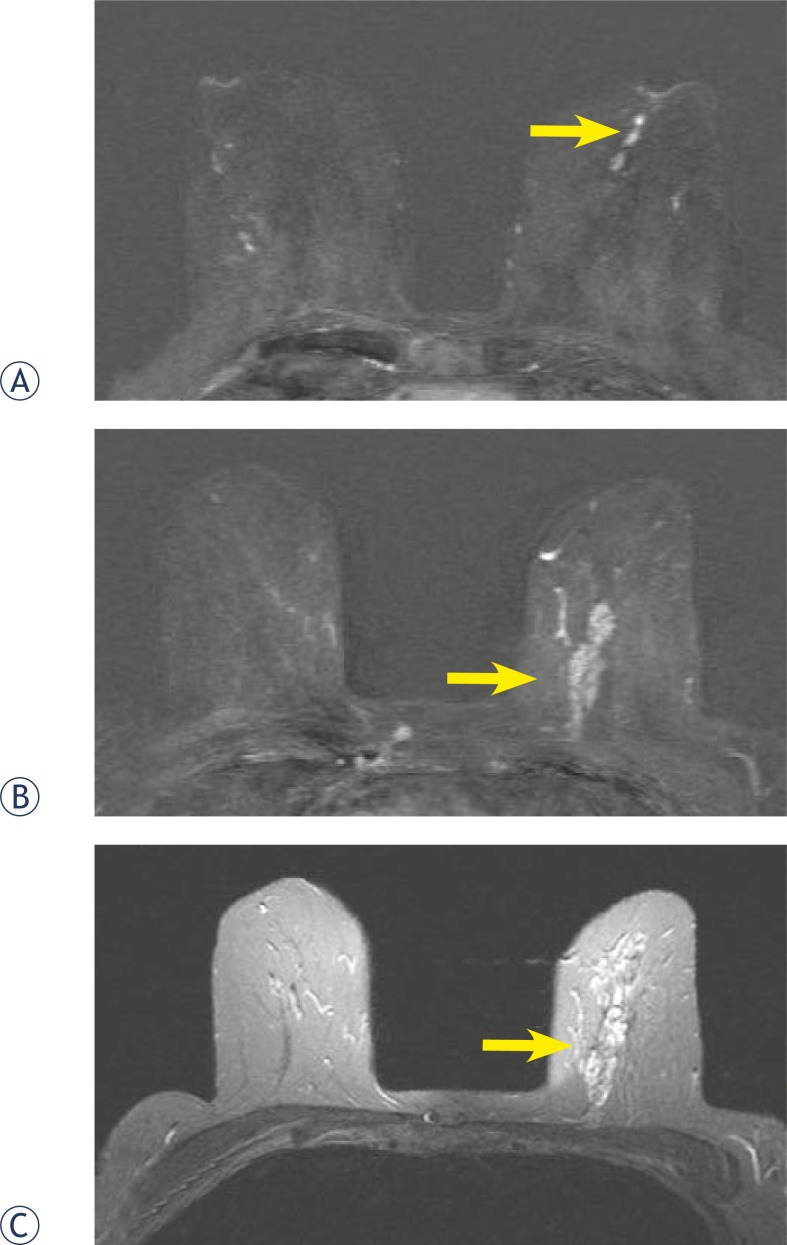
65 year old woman with a history of nipple-discharge in her left breast. Mammographic asimmetry. Ultrasound images showed dilated empty ducts, ductography was suspicious for papilloma. Axial T1-weighted subtracted image after Gadolinium injection (2nd minute) .(a) small round sharply circumscribed masses within a duct sistem–papillomas.(b) segmental, clumped, asymmetric enhancement, fast initial increase and postinitial wash out. (c) high signal intensity on T2–weighted image - BI-RADS 5. Pathologic diagnosis through MRI-guided vacuum-assisted biopsy was massive DCIS with foci of microinvasion.

**FIGURE 6AB.**
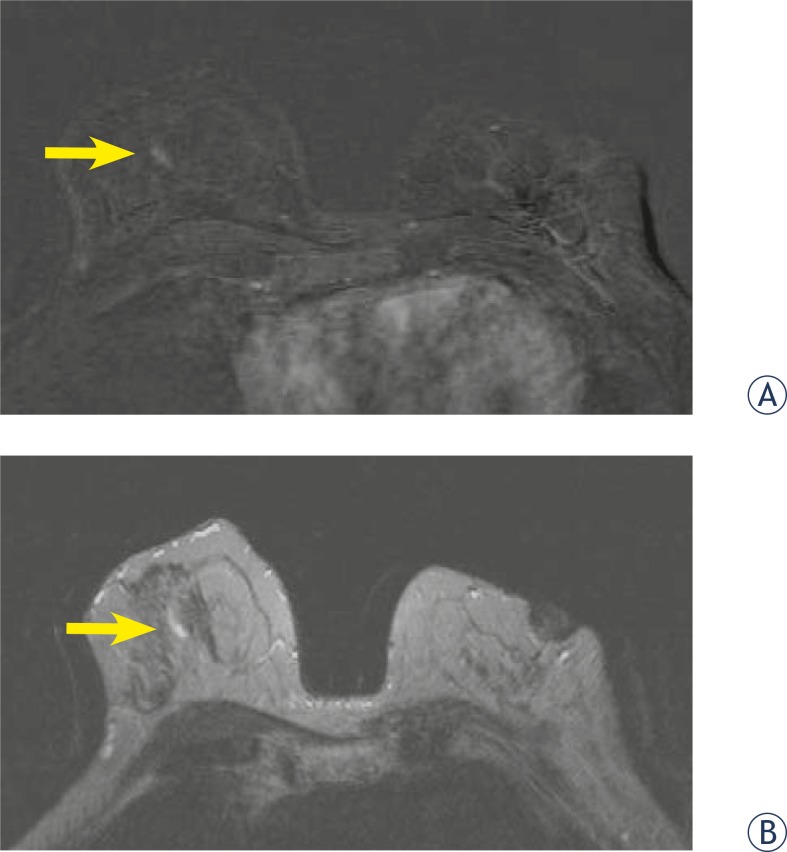
47 year old woman with history of invasive breast carcinoma 10 years ago in her left breast. Retraction of mammila on the right, mammograpic dense breast and benign calcifications. First look and second look ultrasound images showed a cyst. Axial T1-weighted subtracted image after Gadolinium injection (2nd minute).(A) 8×3 mm ductal - linear homogenous, asymmetric enhancement, fast initial increase with postinitial plateau (B) high signal intensity on T2-weighted images, categoriaztion BI-RADS 4. MRI-guided vacuum-assisted biopsy revealed high-grade DCIS withouth microcalcification.

Among these 6 cancers (Figures 1–6) 3 were invasive cancers (2 invasive lobular carcinoma and 1 invasive tubular carcinoma) and other 3 were DCIS (1 massive DCIS with foci of well differentiated invasive carcinoma). Three of 6 cancers were found in women with personal history of breast carcinoma. The median size of the MRI lesions in these 6 cancers was 2.6 cm (range 0.8–6.0 cm). MRI-guided vacuum-assisted biopsy revealed 2 high-risk lesions, in one case there was atypical ductal hyperplasia and in other papilloma. Papilloma was operated on and was proved to be benign.

MRI review suggested that the MRI target might have been excised at vacuum-assisted biopsy in one of these cancers and was sampled in five. Surgery was performed in our institution on 6 of the 6 malignant lesions on 1 of the 2 high-risk lesions and on 1 lesion with discordant result. Finally, 6 of the 14 biopsy lesions were malignant the overall probability of malignancy for an MRI abnormality was 43% (6/14).

The MRI features of the lesions and the probabilities of malignancy according to lesion features are summarized in Table 1. The characteristics of malignant lesions are summarized in Table 2.

## Discussion

MRI-guided-vacuum assisted biopsy has many advantages compared with other biopsy methods for the diagnosis of MRI-detected lesions. In our initial experience with this method, the technical success rate was 93%. In 57% of women MR-VAB yielded benign results and spared most women with MRI-detected lesions the need for the surgical excision. With MR-VAB a continuous suction and acquisition of larger tissue volume is possible. Only MRVAB allows good visualisation of the cavity and/or direct visualisation of the size reduction of the enhancing lesion. It retrieves larger volume of tissue, so we have fewer inadequate specimens. If a lesion is deemed to be not accessible to MR-VAB, MR-guided needle localization followed by surgery can be performed. Our results are in agreement with previous findings in literature.^15^–^19^

MR-VAB can be performed quickly.^20^ Average time to perform biopsy of a single lesion was 39 min in our study. Lesion visibility decreases rapidly over time during the biopsy, due to wash out of contrast in the lesion. The lesion must be identified immediately after the contrast injection. Sampling is dependent on identification and immobilizing the lesion. Lesions that underwent MR-VAB were rated by 2 experienced radiologists in consensus. The BI-RADS MRI lexicon was used. The publication of BI-RADS MRI lexicon in 2003 was an important step toward a standardized approach on the lesion description, but should be regarded as work in progress. Since then some studies have been published about the value of the BI-RADS lexicon and some new predictors for the differentiation of benign from malignant non-mass lesions have been suggested.^21^–^24^ Most of the patients in our series underwent mammography and sonography before MRI-VAB. Only in 3 patients sonography was not performed at the discretion of radiologist. This criteria increased the relative number of non-mass lesions in our study (9/14, 64%) and the number of lesions that are 1cm or smaller in size (6/14, 43%). These lesions are by their nature not well shown by sonography or mammography. Among the cancers, the lesions had non-mass like enhancement characteristics in 5 of 6 cases (83%) and 2 of 6 cancers were smaller than 1cm. Among 5 patients with non-mass like enhancement pattern and proved malignancy, 3 had personal history of breast carcinoma – should be MRI screening a part of an annual follow-up for patients diagnosed with breast cancer.^25^

On T2-weighted images one cancer showed low signal intensity, three intermediate and 2 high signal intensities. Among 5 non-mass like enhancing lesions there was one lesion with low signal intensity on T2 weighted image, 2 lesions with intermediate and 2 lesions with high signal on T2 weighted images. Both lesions with high-signal were DCIS, one with ductal, homogenous enhancement smaller than 1 cm and the other one with segmental clumped enhancement 6 cm in size. As published in literature, the permeability of the basement membrane of DCIS-containing ducts, is increased, allowing gadolinium chelates to penetrate the membrane and accumulate within the DCIS-filed milk ducts. That is proposed to be the explanation for enhancement of purely DCIS on breast MR Images.^26^–^31^

In some cases, after MRI-VAB, benign histology offers no explanation for a contrast-enhancing lesion. The radiologic/pathologic mismatch is more problematic for MRI-VAB than for mammographically-guided interventions because no specimen radiography can be used to verify appropriate core biopsy. If any doubt persists, early post-biopsy breast MRI is performed in our practice but not earlier than after 6 months.^32^ There were 2 cases, where after 6-months follow up the lesions were unchanged in size and morphology. In one case the surgical excision revealed radial scar. In the other one re-biopsy was done and the lesion proved to be benign.

Our study has some limitations. Our series is small and represents our initial experience. Because this study is retrospective, the lesion descriptions in a few cases did not use terms from the BI-RADS MRI lexicon, thus, we modified them as much as possible. Two patients underwent MRI before biopsy at other institution and we could not analyze morphologic and kinetics, only the reports were available. In one case, where discordant findings were 4 showed, we had incomplete follow-up data.

In conclusion, MRI-guided vacuum-assisted breast biopsy in our initial experience proved to be a fast, save and accurate method. The cancer rate of women who underwent MR-VAB was 43% in our cohort. The morphologic pattern of non-mass like enhancement and washout or plateau kinetics showed a higher cancer rate than mass like enhancement and persistent kinetics.

## Figures and Tables

**FIGURE 7:**
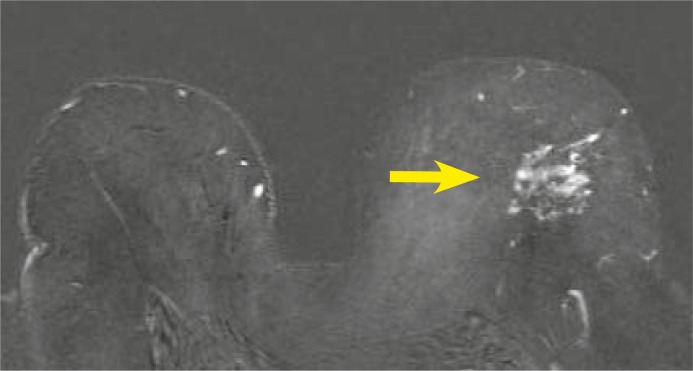
64-year old woman with history of invasive breast carcinoma in her right breast 10 years ago. Mammography and ultrasound images showed discrete architectural distortion in the left breast. US-guided core biopsy was inconclusive. Axial T1-weighted subtracted image after Gadolinium injection (2^nd^ minute) shows regional, heterogeneous, asymmetric, non-mass like enhancement, fast initial increase, post initial wash-out, intermediate signal intensity on T2-weighted images - BI-RADS 5. Pathologic diagnosis through MRI-guided vacuum-assisted biopsy was DCIS.

**FIGURE 8:**
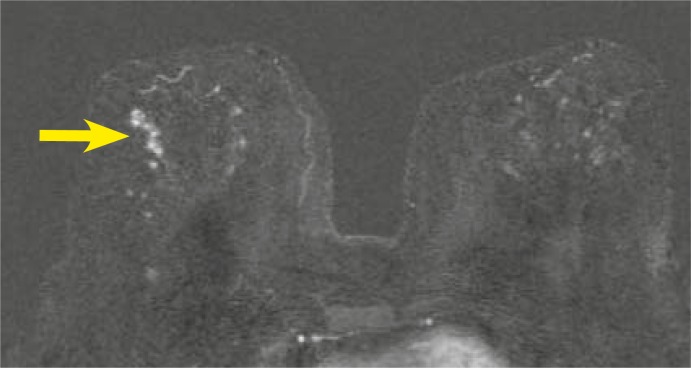
57-year old woman, US-guided fine needle biopsy performed at another institution was suspicious for pappilary carcinoma. Mammographically dense breast, first and second look ultrasound images at institute of oncology showed simple cyst. Axial T1-weighted subtracted image after. Gadolinium injection (2^nd^ minute) shows ductal, reticular-dendritic, asymmetric, non-mass like enhancement, fast initial enhancement with post initial plateau. Intermediate signal intensity on T2-weighted images. BI-RADS 4. MRI-guided vacuum-assisted biopsy revealed invasive tubular carcinoma.

**FIGURE 9:**
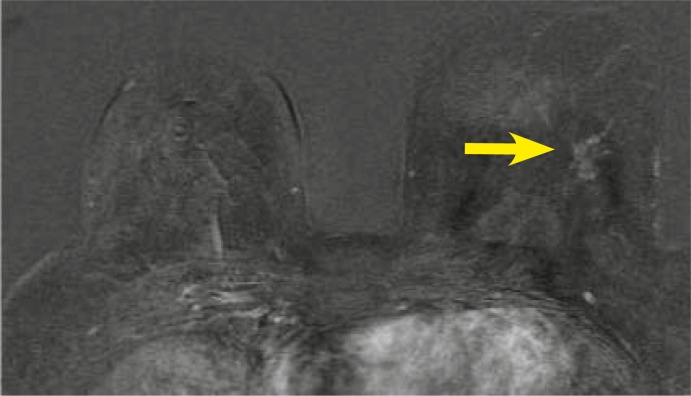
63-year old woman with history of invasive ductal carcinoma in her right breast 8 years ago. Clinical exam revealed enlarged lymph node in the left axilla. Fine needle aspiration biopsy showed metastatic lymph node. Mammographically discrete architectural distortion only in one projection in the left and postoperative changes in the right breast. Ultrasound was not performed at the discretion of the radiologist. Axial T1-weighted subtracted image after Gadolinium injection (2^nd^ minute) shows focal, heterogeneous, asymmetric, non–mass like enhancement in the left breast, the kinetic curve shows fast initial enhancement with post-initial wash out, low signal intensity on T2-weighted images – BI-RADS 4. MRI–guided vacuum-assisted biopsy revealed invasive lobular carcinoma.

**FIGURE 10:**
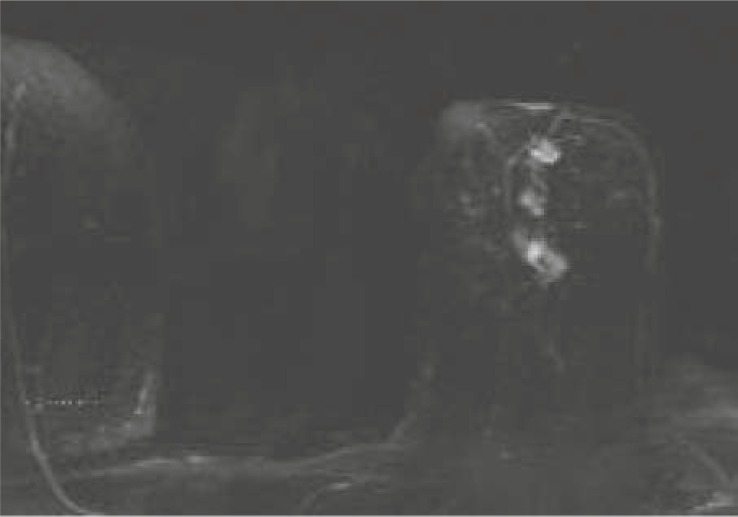
71-year old woman, MR mammography performed in the other institution. Axial T1-weighted subtracted image after Gadolinium injection (2nd minute) shows 3 round masses in her left breast, with spiculated margins, rim like enhancement, intermediate signal intensity on T2-weighted images. Kinetic curve shows fast initial enhancement with post-initial wash out. Lesions were categorized as BI-RADS 5. Pathologic diagnosis through MRI-guided vacuum-assisted biopsy was invasive lobular carcinoma.

**TABLE 1:** MRI findings of 14 targeted lesions and the probability of malignancy

**Features**	**No. of Lesions/Total No. of Lesions (%)**	**No. of Malignant Lesions/No. of Lesions (%)**
**Lesion type**		
Focus	0	0
Mass	5/14 (36)	1/5 (20)
Nonmass	9/14 (64)	5/9 (55)
Total	14/14 (100)	6/14 (43)

**Morphology**		

**Margin of mass**		

Smooth	2/14 (14)	0/2 (0)
Nonsmooth	3/14 (21)	1/3 (33)

**Distribution of Nonmass**		

Regional	1/14 (7)	1/1 (100)
Segmental	1/14 (7)	1/1 (100)
Ductal	6/14 (43)	2/6 (33)
Focal areas	1/14 (7)	1/1 (100)
Total	9/14 (64)	5/9 (55)

**Kinetic feature**		

Persistent	1/14 (7)	0/1 (0)
Plateau	6/14 (43)	2/6 (33)
Washout	7/14 (50)	4/7 (57)
Total	14/14 (100)	6/14 (43)

**Signal intensity T2-weigted images**		

High	4/14 (23)	2/4 (50)
Intermediate	8/14 (57)	3/8 (37)
Low	2/14 (14)	1/2 (50)
Total	14/14 (100)	6/14 (43)

**TABLE 2:** The characteristics of the malignant lesions

**Site of the lesion**	Right	2
	Left	4

**Type of the lesion**	MLE	1
	NMLE	5

**T2-weighted images Signal intensity**	High	2
	Intermediate	3
	Low	1

**Kinetics**	Rapid-plateau	2
	Wash-out	4

**Size**	2.6cm (range; 0.8 – 6)	

**Histologic type Of cancer**	Invazive lobular	2
	Invazive tubular	1
	DCIS	3

MLE = masslike enhancemnt; NMLE = nonmasslike enhancement; DCIS = ductal carcinoma in situ

## References

[1] Podobnik J, Kocijancic I, Kovac V, Sersa I (2010). 3T MRI in evaluation of asbestos-related thoracic diseases - preliminary results. Radiol Oncol.

[2] Stanic K, Kovac V (2010). Prophylactic cranial irradiation in patients with small-cell lung cancer: the experience at the Institute of Oncology Ljubljana. Radiol Oncol.

[3] Kuhl CK, Schrading S, Bieling HB, Wardelmann E, Leutner CC, Koenig R (2007). MRI for diagnosis of pure ductal carcinoma in situ: a prospective observational study. Lancet.

[4] Lehman CD, Deperi ER, Peacock S, McDonough MD, Demartini WB, Shook J (2005). Clinical experience with MRI-guided vacuum-assisted breast biopsy. AJR Am J Roentgenol.

[5] Lehman CD, DeMartini W, Anderson BO, Edge SB (2009). Indications for breast MRI in the patient with newly diagnosed breast cancer. Indications for breast MRI in the patient with newly diagnosed breast cancer. J Natl Compr Canc Netw.

[6] Sardanelli F, Podo F, D’Agnolo G, Verdecchia A, Santaquilani M, Musumeci R (2007). Multicenter comparative multimodality surveillance of women at genetic-familial high risk for breast cancer (HIBCRIT study): interim results. Radiology.

[7] Liberman L, Mason G, Morris EA, Dershaw DD (2006). Does size matter? Positive predictive value of MRI-detected breast lesions as a function of lesion size. AJR Am J Roentgenol.

[8] Warner E, Plewes DB, Hill KA, Causer PA, Zubovits JT, Jong RA (2004). Surveillance of BRCA1 and BRCA2 mutation carriers with magnetic resonance imaging, ultrasound, mammography, and clinical breast examination. JAMA.

[9] Sardanelli F, Boetes C, Borisch B, Decker T, Federico M, Gilbert FJ (2010). Magnetic resonance imaging of the breast: recommendations from the EUSOMA working group. Eur J Cancer.

[10] Heywang-Köbrunner SH, Heinig A, Schaumlöffel U, Viehweg P, Buchmann J, Lampe D (1999). MR-guided percutaneous excisional and incisional biopsy of breast lesions. Eur Radiol.

[11] Prat X, Sittek H, Grosse A, Baath L, Perlet C, Alberich T (2002). European quadricentric evaluation of a breast MR biopsy and localization device: technical improvements based on phase I evaluation. Eur Radiol.

[12] Tozaki M, Yamashiro N, Fukuma E (2007). MR-guided vacuum-assisted breast biopsy using a non-titanium needle. Magn Reson Med Sci.

[13] Perlet C, Heywang-Kobrunner SH, Heinig A, Sittek H, Casselman J, Anderson I (2006). Magnetic resonance-guided, vacuum-assisted breast biopsy: results from a European multicenter study of 538 lesions. Cancer.

[14] Heywang-Köbrunner SH, Sinnatamby R, Lebeau A, Lebrecht A, Britton PD, Schreer I (2009). Consensus Group Interdisciplinary consensus on the uses and technique of MR-guided vacuum-assisted breast biopsy (VAB): results of a European consensus meeting. Eur J Radiol.

[15] Heywang-Köbrunner SH, Schaumlöffel-Schulze U, Heinig A, Beck RM, Lampe D, Buchmann J (1999). MR-guided percutaneous vacuum biopsy of breast lesions: experiences with 100 lesions. [Abstract]. Radiology.

[16] Liberman L, Bracero N, Morris E, Thornton C, Dershaw DD (2005). MRI-guided 9-gauge vacuum-Assisted breast biopsy: initial clinical Experience. AJR Am J Roentgenol.

[17] Orel SG, Rosen M, Mies C, Schnall MD (2006). MR imaging-guided-9-gauge vasuum-assisted core-needle breast biopsy: initial experience. Radiology.

[18] Perlet C, Heywang-Kobrunner SH, Heinig A, Sittek H, Casselman J, Anderson I (2006). Magnetic resonance-guided, vacuum-assisted breast biopsy: results from a European multicenter study of 538 lesions. Cancer.

[19] Tozaki M, Yamashiro N, Sakamoto M, Sakamoto N, Mizuuchi N, Fukuma E (2010). Magnetic resonance-guided vacuum-assisted breast biopsy: results in 100 Japanese women. Jpn J Radiol.

[20] Noroozian M, Gombos EC, Chikarmane S, Georgian-Smith D, Raza S, Denison CM (2010). Factors that impact the duration of MRI-guided core needle biopsy. AJR Am J Roentgenol.

[21] American College of Radiology (2003). ACR BI-RADS MRI. ACR Breast Imaging Reporting and Data System, breast imaging atlas.

[22] Kuhl CK, Shild HH, Morakkabati N (2005). Dynamic bilateral contrast-enhanced MR imaging of the breast: trade-off between spatial and temporal resolution. Radiology.

[23] Goto M, Ito H, Akazawa K, Kubota T, Kizu O, Yamada K (2007). Diagnosis of breast tumors by contrast-enhanced MR imaging: comparison between the diagnostic performance of dynamic enhancement patterns and morphologic features. J Magn Reson Imaging.

[24] Sohns C, Scherrer M, Staab W, Obenauer S (2011). Value of the BI-RADS Classification in MR-mammography for diagnosis of benign and malignant breast tumors. European Radiology.

[25] Zebic-Sinkovec M, Kadivec M, Podobnik G, Skof E, Snoj M (2010). Mammographycally occult high grade ductal carcinoma in situ (DCIS) as second primary breast cancer, detected with MRI, a case report. Radiol Oncol.

[26] Baltzer PA, Dietzel M, Kaiser WA (2011). Nonmass lesions in magnetic resonance imaging of the breast: additionhal T2-weigted images improve diagnostic accuracy. J Comput Assist Tomogr.

[27] Jansen SA, Paunesku T, Fan X, Woloschak GE, Vogt S, Conzen SD (2009). Ductal carcinoma in situ: x-ray fluorescence microscopy and dynamic contrast-enhanced MR imaging reveals gadolinium uptake within neoplastic mammary ducts in a murine model. Radiology.

[28] Kuhl CK (2009). Why do purely intraductal cancers enhance on breast MR Images?. Radiology.

[29] Mossa-Basha M, Fundaro GM, Shah BA, Ali S, Pantelic MV (2010). Ductal carcinoma in situ of the breast: MR imaging findings with histopathologic correlation. Radiographics.

[30] Newstead GM (2010). MR Imaging of ductal carcinoma in situ. Magn Reson Imaging Clin N Am.

[31] Miklavcic D, Towhidi L (2010). Numerical study of the electroporation pulse shape effect on molecular uptake of biological cells. Radiol Oncol.

[32] Li J, Dershaw DD, Lee CH, Kaplan J, Morris EA (2009). MRI follow-up after concordant, histologically benign diagnosis of breast lesions sampled by MRI-guided biopsy. AJR Am J Roentgenol.

